# Adaptive Response Behavior in the Pursuit of Unpredictably Moving Sounds

**DOI:** 10.1523/ENEURO.0556-20.2021

**Published:** 2021-05-04

**Authors:** José A. García-Uceda Calvo, Marc M. van Wanrooij, A. John Van Opstal

**Affiliations:** Donders Centre for Neuroscience, Department of Biophysics, Radboud University, Nijmegen 6525 AJ, The Netherlands

**Keywords:** auditory fovea, auditory motion perception, head movement, human, linear systems, sound localization

## Abstract

Although moving sound-sources abound in natural auditory scenes, it is not clear how the human brain processes auditory motion. Previous studies have indicated that, although ocular localization responses to stationary sounds are quite accurate, ocular smooth pursuit of moving sounds is very poor. We here demonstrate that human subjects faithfully track a sound’s unpredictable movements in the horizontal plane with smooth-pursuit responses of the head. Our analysis revealed that the stimulus–response relation was well described by an under-damped passive, second-order low-pass filter in series with an idiosyncratic, fixed, pure delay. The model contained only two free parameters: the system’s damping coefficient, and its central (resonance) frequency. We found that the latter remained constant at ∼0.6 Hz throughout the experiment for all subjects. Interestingly, the damping coefficient systematically increased with trial number, suggesting the presence of an adaptive mechanism in the auditory pursuit system (APS). This mechanism functions even for unpredictable sound-motion trajectories endowed with fixed, but covert, frequency characteristics in open-loop tracking conditions. We conjecture that the APS optimizes a trade-off between response speed and effort. Taken together, our data support the existence of a pursuit system for auditory head-tracking, which would suggest the presence of a neural representation of a spatial auditory fovea (AF).

## Significance Statement

Inspired by the visual ocular smooth-pursuit system, several studies have used eye movements to track moving sounds, but obtained poor pursuit performance, which led to the idea that the auditory system lacks sensitivity to sound velocity. We here demonstrate accurate head-pursuit of sounds, moving along unpredictable trajectories in the horizontal plane. Interestingly, the auditory pursuit responses adapted to the covert movement spectrum of the stimulus ensemble, from which we infer that the system may optimize a trade-off between movement speed and effort. Our results support the existence of an auditory pursuit system (APS), and we discuss its implications for the neural mechanisms that represent and track moving sounds.

## Introduction

To infer source directions in the horizontal plane of the head, the auditory system extracts interaural differences in arrival time and sound level [interaural level differences (ITDs) and interaural timing differences (ILDs), respectively; Middlebrooks and Green, 1991; Blauert, 1997]. Front-back and up-down localization relies on the interaction of sound-waves within the pinnae, resulting in idiosyncratic direction-dependent spectral acoustic filters (Musicant and Butler, 1984; Oldfield and Parker, 1984; Wightman and Kistler, 1989; Middlebrooks, 1992; Hofman et al., 1998; Van Wanrooij and Van Opstal, 2005).

However, auditory scenes typically contain moving sounds, and subjects may move actively or passively through the environment. As accurate sound-motion perception would enable the prediction of sound-source trajectories in the environment (Crum and Hafter, 2008), neural processing of dynamic acoustic-cue changes is crucial to track moving sounds (Vliegen et al., 2004).

Perceptual sensitivity to acoustic motion has been quantified by the minimum audible movement angle (Mills, 1958; Harris and Sergeant, 1971; Grantham, 1986). Sound-motion perception has typically been studied with the head stationary, inspired by studies of visual-motion mechanisms. An unresolved issue is whether moving sounds are processed by neural mechanisms tuned to continuous motion, or by a snapshot position-localization mechanism.

Moving visual targets are tracked with smooth-pursuit eye movements (Rashbass, 1961; Robinson, 1965; Krauzlis and Lisberger, 1994; Krauzlis, 2004; Barnes, 2008; Lisberger, 2010). Visual feedback provides the positional error and retinal slip velocity, needed to realign the fovea with the target through corrective saccades and smooth pursuit. Because of significant visual-motor delays (≈80 ms; Robinson, 1965), visual feedback alone is insufficient for accurate pursuit, which also incorporates higher-level predictive mechanisms (Barnes, 2008). Neurons in visual-cortical motion areas like MST encode the direction and velocity of foveal stimuli and underlie the generation of accurate smooth-pursuit eye movements (Mikami et al., 1986; Dürsteler et al., 1987; Krauzlis and Lisberger, 1991; Ilg and Thier, 2008).

The question whether similar mechanisms exist in the auditory system has so far received little attention. Neurons in inferior colliculus (IC) and medial geniculate nucleus (cat: Al’tman et al., 1985; Bekhterev, 2003; guinae pig: Ingham et al., 2001; bat: Olsen and Suga, 1991; Pollack, 2012; barn owl: Wagner and Takahashi, 1992), and in auditory cortex (human EEG: Kreitewolf et al., 2011; cat: Stumpf et al., 1992; Toronchuk et al., 1992; Poirier et al., 1997; rat: Doan and Saunders, 2003; bat: Firzlaff and Schuller, 2001), have been shown to be sensitive to simulated dichotic sound motion. However, there is no conclusive evidence yet for an active auditory pursuit mechanism.

Brief sounds can elicit accurate goal-directed eye movements (Hofman and Van Opstal, 1998). Yet, smooth eye movements to moving sounds are practically non-existent, as ocular sound-tracking occurs through a series of saccades, with at best low-gain smooth-pursuit (Boucher et al., 2004; Berryhill et al., 2006). This led to the hypothesis that sound motion is perceptually tracked by intermittent sampling of the source position (Middlebrooks, 2015; Carlile and Leung, 2016), rather than by a continuous measurement of sound velocity.

However, as ocular sound-tracking does not affect any acoustic localization or pursuit error, we reasoned that, instead, an appropriate head-tracking response could keep craniocentric acoustic cues near the region of highest spatial acuity, just like in visual pursuit. So far, only few studies have investigated auditory-evoked head-tracking. For example, cats tracked apparent-motion clicks through multiple head-saccades, as may be expected from a snap-shot mechanism (Beitel, 1999; Middlebrooks, 2015), and human head-tracking in a virtual-reality setup indicated that tracking accuracy degraded at higher simulated sound-velocities (Carlile and Leung, 2016).

Self-generated head movements facilitate the externalization of virtual-reality sounds (Brimijoin et al., 2013), which suggests a tight integration of the sound-localization cues with neural motor commands, and emphasizes the importance of sensorimotor integration in sound localization (Makous and Middlebrooks, 1990; Vliegen et al., 2004; Pallus and Freedman, 2016; Van Opstal, 2016). In line with this notion, it was recently demonstrated that active head movements significantly improve acoustic distance perception (Genzel et al., 2018). Such a sensorimotor relationship has so far not been studied for auditory pursuit under free-field hearing conditions.

In this article, we therefore characterized human head-movement pursuit to a free-field sound, moving along unpredictable trajectories in the horizontal plane. Listeners only had access to the acoustic input, and their self-generated head movements.

**Figure 1.**
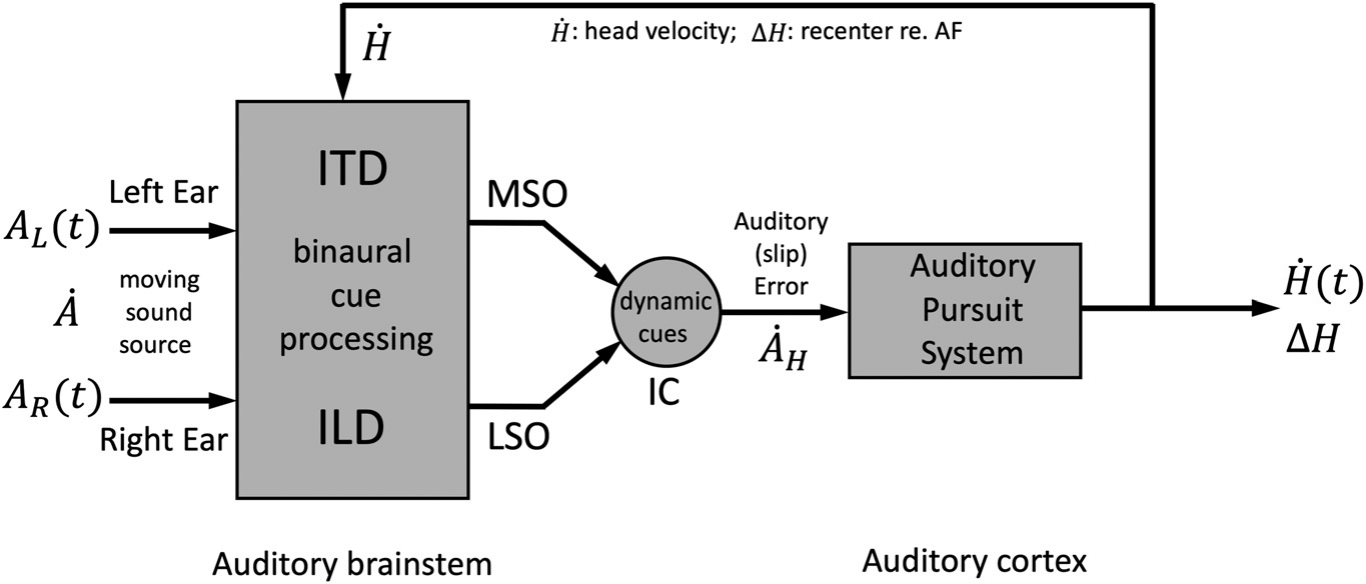
Presumed processing stages for horizontal auditory head pursuit. Both ears receive time-varying ILD and ITD because of a moving sound in the horizontal plane, and the head turning at angular velocity $\dot{H}$. Integration of these dynamic cues provides an estimate of sound velocity with respect to the head, ${\dot{A}}_{H}\left(t\right)$ (auditory slip velocity). The APS aims to minimize the auditory slip-velocity error and the source-position error, ΔH, with respect to the Auditory Fovea (AF), by bringing and keeping the instantaneous ILD and ITD cues close to zero.

The rationale of our study is illustrated by the scheme in Figure 1. We hypothesized that, like for visual pursuit, accurate head tracking of the sound source requires an auditory pursuit system (APS) that would be driven by an auditory slip error. This error arises, because of an ongoing difference in sound and head velocity, and because the head-centered sound-location may differ with respect to a head-fixed auditory fovea (AF). The AF would represent the region of highest spatial acuity, and is presumably located around the straight-ahead direction, where the ILDs and ITDs are close to zero and have their highest resolution (Mills, 1958). Note that in contrast to the visual fovea in the retinae of both eyes, the representation of an AF would result from a neuro-computational mechanism, as it results from binaural integration. The auditory slip-error with respect to the AF, ${\dot{A}}_{H}\left(t\right)$, results from the difference between sound velocity relative to the head, $\dot{A}$, and head velocity, $\dot{H}$, and the localization error, ΔH, which may all be derived from the dynamic changes in acoustic ITD/ILD cues in the auditory midbrain IC. A recentering (saccadic) head movement, ΔH, would bring the sound close to the AF, so that when head velocity and position equal sound velocity and position, medial superior olive (MSO) and lateral superior olive (LSO) will both signal a (near-)zero ILD/ITD.

## Materials and Methods

### Subjects

Eleven subjects (S1–S11; five females; ages 21–43 years) participated in the experiments after providing their informed consent. All subjects had normal binaural hearing, had no motor problems, and normal or corrected-to-normal vision. The first author of this study was one of the participants. All other subjects were not aware of the purpose of the study. Five subjects had participated in other sound-localization experiments in the laboratory. To get familiarized with the experimental procedures, the naive subjects first received a short practice session before the actual experiments.

### Ethics

The experiments fully adhered to the protocols regarding observational experiments on healthy human adults and were approved by the local institutional ethical committee of the Faculty of Social Sciences (ECSW 2016-2208-41). All participants signed an informed consent form, before the start of the experimental sessions.

### Experimental setup

Subjects were seated in a completely dark anechoic chamber (3 × 3 × 3 m^3^) in which the background noise level was ∼30-dB SPL (A-weighted). Reflections above 500 Hz were effectively absorbed by black radio-absorbent material (UXEM Flexible Foams) that was mounted on the floor, walls, ceiling and on every large object present in the room.

**Figure 2.**
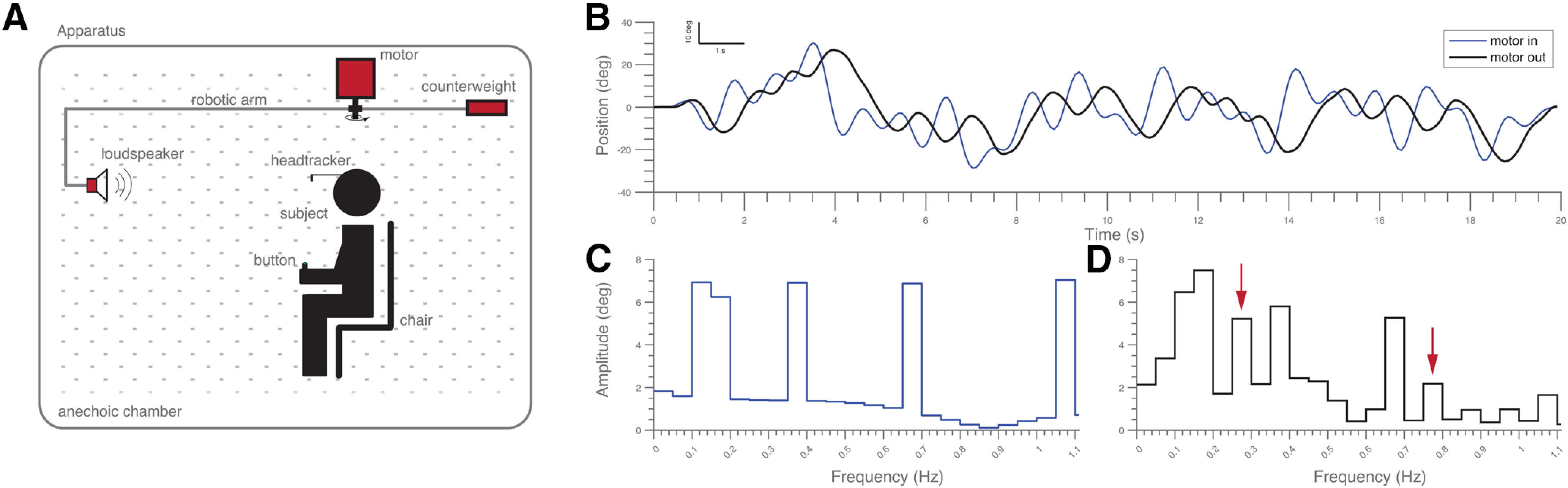
Input-output transformation of the robot arm and setup. ***A***, Schematization of the experimental set-up. The subject sits erect on a comfortable chair in the center of a circle described by the custom-made light-weight robot arm. The motor driving the robot arm was fixed at the subject’s zenith. The target sound emanated from the loudspeaker, which was mounted at the end of the robotic arm that horizontally rotated the speaker in a pseudo-randomly selected direction (Eq. 2) along a circular trajectory (radius, 1.15 m). ***B–D***, Input-output traces [programmed stimulus, ${\hat{\alpha }}_{n}\left(t\right)$, blue, Eq. 2; and motor movement, m_n_(t), black] and the associated amplitude spectra of the input and output signals. Note that the robot arm added some additional frequency components (red arrows point to examples) to the programmed stimulus.

The auditory stimuli were presented from a broadband loudspeaker (SC5.9, Visaton; Art. No. 8006) mounted on a custom-made L-shaped robotic arm that was driven by a DC motor (JVL MAC140-A1 integrated servomotor, Gearbox Wittenstein Alpha–Angular Hollow Shaft MF2-50-5B1). The input signal for the motor was programmed in MATLAB (The MathWorks) and sent to the DC motor through a Tucker Davis Technologies TDT-RP2.1 ADC module. This setup (Fig. 2*A*) enabled rapid and accurate positioning of the speaker at a fixed distance of 1.15 m at any azimuthal direction around the subjects’ head.

Head orientation and actual speaker movements were measured with a magnetic search-coil system (Robinson, 1963). Briefly, three orthogonal magnetic fields were generated by alternating currents of three different frequencies passing through three pairs of 3 × 3 m^2^ squared coils, spanned along the edges of the room, which in turn induced alternating voltages in a small search coil (diameter, ∼5 cm) attached on a light-weight glasses frame that was adjusted to fit on the subjects’ head, without interfering with the ears. This system enabled accurate recording of 3D head orientations at a resolution of 0.1°, or better (Van Bentum et al., 2017).

From the center of the glasses frame, a 40 cm long, thin, aluminum rod (weight ∼50 g) protruded forward with a small 1-cm^2^ black plate attached to its end, which was positioned in front of the subjects’ eye, and on which a dim red laser spot was projected from the subject’s nose bridge. The laser spot served as a head-fixed and eye-fixed pointer, and helped the subject to fixate the eye-in-head orientation, while pointing with the head to the sound source. This procedure thus ensured accurate measurement of sound-evoked head movements without the co-occurring saccadic eye movements of natural gaze shifts.

### Stimulus characteristics

Auditory stimuli consisted of Gaussian white noise with a duration of 20 s. Sounds had 5-ms sine-squared and cosine-squared onset and offset ramps, and a flat spectrum (within 2 dB) within their pass band between 0.5 and 20 kHz, and were digitally generated in MATLAB. The signals were sent to a real-time processor (RP2.1 System3; Tucker-Davis Technologies) at a sampling rate of 48 to 828.25 Hz. After attenuation by custom-built amplifiers, the audio signal was sent to the loudspeaker, which was moved in a pseudo-random, unpredictable direction (clockwise and anticlockwise). Stimulus coordinates ranged from −30° to +30° in azimuth, and at 0° in elevation. All stimuli were well discernible, and kept at a fixed intensity level of 55-dB SPL (A-weighted). Absolute free-field sound levels were measured, with the Brüel & Kjær BK2610 sound amplifier and a Brüel & Kjær BK4144 microphone, at the location of the subject’s head.

The buzzing sounds coming from the activated motor were near 50 dBA at the subject’s ears, and always came from the subject’s zenith, 90° away from the horizontal plane. These sounds did not provide any cue about stimulus location or direction. We tested this qualitatively while the motor was activated, but without playing a target sound. When the target sound played, the motor sounds did not interfere with the listeners’ sound-localization abilities.

The programmed sound-source movements consisted of a linear combination of five sines, digitally generated and stored as a wav-file in MATLAB, to be subsequently sent as a command movement to the robotic arm. Stimulus generation was performed as follows. First, the dynamic sound-source locations, $\alpha_{n}(t)$, for stimulus, n, with *n* = [1:30], were defined as:

(1) $$\alpha_{n}\left(t\right)=\overset{5}{\underset{k=1}{\sum }}A\cdot \sin\left(2\pi tf_{k}+\varphi_{n,k}\right).$$

The frequency components, $f_{p}$, were fixed multiples of the fundamental frequency, *f_0_* = 0.05 Hz, i.e., *f_p_* = *p* × *f_0_*, with *p* = [2,3,7,13,21]. The stimuli thus had a period that corresponded to the total trial length of 20 s.

Each component in Equation 1 had a constant amplitude, *A*, while its phase, $\varphi_{n,k}$, was selected at random between [0,2 π]. Because of the latter, the maximum amplitude of *α_n_(t)* changed too. We therefore normalized each stimulus by its peak amplitude to a peak excursion of 30°, which resulted in a pseudorandom trial-to-trial variation of the component amplitudes, *A_k_ = A/*max(|*α_n_(t)*|), of the harmonics in the stimuli. In this way, the stimulus movements were unpredictable for the subject.

The actual robot movements (i.e., the subjects’ true stimulus motion), *m_n_(t)*, were measured with a search coil attached to the speaker, and resulted in a slightly nonlinear, and filtered, transformation, *h(α)*, of the command input (Eq. 1) to the robotic arm. The true stimulus motion of the motor, *m_n_(t)*, was thus described by the following:

(2) $${\hat{\alpha }}_{n}\left(t\right)=\overset{5}{\underset{k=1}{\sum }}A_{k}\cdot \sin\left(2\pi tf_{k}+\varphi_{n,k}\right)and\;m_{n}\left(t\right)=h\left[{\hat{\alpha }}_{n}\left(t\right)\right].$$

Figure 2 shows the robot’s response for one of the stimuli, and the associated frequency components that resulted from the stimulus-to-movement transformation, *h*. Note that because of the nonlinear characteristic of *h*(α*)*, the actual motion of the speaker could contain some additional harmonics, e.g., at *p* = [1,5,15], i.e., at 0.05, 0.25, and 0.75 Hz.

### Psychophysics

Subjects performed two psychophysical tasks in different sessions. Both sessions started with a calibration procedure for the head-mounted coil. The first session assessed baseline sound-localization performance in the azimuth and elevation directions, by means of a standard sound-localization task, consisting of 150 trials. In the second session, the subject performed the auditory pursuit paradigm. The latter consisted of thirty different trials of 20 s each. To prevent fatigue that would potentially degrade performance, this session lasted ∼25 min. The localization and pursuit tasks were executed under open-loop conditions, in darkness, and without any kind of verbal or visual feedback. For safety reasons, the subject was observed by the experimenter through an infrared camera that was placed in the experimental room.

### Calibration procedure

To obtain the head-position data for the calibration procedure, the subject accurately pointed the head-fixed laser pointer (see above, Experimental setup) toward 56 LED locations distributed over the two-dimensional frontal hemifield. A feedforward three-layer neural network was trained to map the measured endpoints into degrees azimuth and elevation of the LEDs. This neural network was subsequently used for offline calibration of the head-coil signals obtained from the subjects’ head-movement responses to the auditory stimuli in the localization task and pursuit experiment.

### Static sound-localization task

To measure the baseline sound-localization performance of the subjects, a standard sound-localization task was performed. The subjects were instructed to point at a straight-ahead fixation LED and push a button whenever they felt ready. After the button press, the fixation LED extinguished, and ∼200 ms later an auditory Gaussian white noise burst with a duration of 150 ms was presented at a fixed intensity of 55 dBA at a randomly selected location in the subjects’ two-dimensional frontal hemifield. The subjects had to point the head, as quickly and as accurately as possible, to the perceived sound location.

### Auditory pursuit task

Subjects were instructed to point at a straight-ahead fixation LED and subsequently pushed a button whenever they felt ready. Immediately after the button press, the fixation LED went off, and ∼200 ms later the auditory stimulus, consisting of 20 s continuous Gaussian white-noise, appeared at an intensity of 55 dBA. As soon as the stimulus was heard, the subject had to point and track the sound source with the head-mounted-LED (which was continuously on), as accurately as possible.

### Data analysis

All data analysis procedures were performed in MATLAB R2018b (The MathWorks). The coordinates of the moving sound and the head-movement responses were expressed in the double-pole azimuth-elevation coordinate system, in which the origin coincides with the center of the head (Knudsen and Konishi, 1979). The analysis of head movements was performed offline with custom-made software that automatically detected head displacements and saccades in the calibrated data. Detected movements and saccades were checked visually without stimulus information to the experimenter, and onset and offsets could be corrected manually, if needed.

### Sound localization

We quantified the static sound-localization performance of each subject by linear regression on the stimulus-response relations for azimuth and elevation:

(3) $$\alpha_{r}=b\cdot \alpha_{t}+a\;and\;\varepsilon_{r}=d\cdot \varepsilon_{t}+c,$$

with $\alpha_{r}$, $\alpha_{t}$, $\varepsilon_{r}$, and $\varepsilon_{t}$ the response azimuth, target azimuth, response elevation, and target elevation, respectively. Fit parameters, a and c, are the response biases (offsets, in degrees), whereas b and d are the response gains (slopes, dimensionless) of the azimuth and elevation responses. The parameters a,b,c,d were found by minimizing the mean-squared error (MSE) of Equation 3 (Press et al., 1992). From each linear fit, we also determined the correlation coefficient between data and fit, the mean absolute error, and the SD of the residuals of the responses.

We verified normal localization performance in azimuth and elevation of all subjects, with gains close to 1.0, biases close to 0°, and high correlation coefficients, typically exceeding 0.9; here, we will not report further on the results of these standard control localization experiments.

### Modelling the pursuit responses

Sound-source pursuit in the horizontal plane was quantified by the frequency content of the stimulus-response waveforms. During pursuit, subjects did not make appreciable vertical head movements. To compare the significant frequency components in the stimuli with those present in the subjects’ responses we applied the fast Fourier transform to the stimulus and response signals. From the resulting spectra, we determined the gain-shift and phase-shift characteristics of the responses with respect to the measured stimulus movement for each trial.

We subsequently modelled the pursuit transfer characteristic of each trial, *n*, by a second-order linear filter, in series with a delay, *T_D_*, as has been done before for the ocular visual-pursuit system (Krauzlis and Lisberger, 1994; Barnes, 2008). In the Laplace domain, the transfer characteristic of the APS, *H_APS,n_(s)*, is then given by the following:

(4) $$H_{APS,n}\left(s\right)=G_{0}\cdot \text{exp}\left(-T_{D}\cdot s\right)\cdot \frac{\omega_{C,n}^{2}}{\omega_{C,n}^{2}+2\zeta_{n}\omega_{C,n}s+s^{2}},$$

with $\omega_{C,n}=2\pi f_{C}$ the angular resonance frequency of the (undamped) system, $\omega_{C,n}=2\pi /T_{C,n}$ (with *T_C,n_ = 1/f_C_* the system’s undamped time constant), *G_0_* the system’s steady-state gain (at s = 0), and ζ_n_ the system’s damping ratio (dimensionless). The delay, $T_{D}$, was determined by brute-force fitting, and clamped at a fixed value, separate for each subject (values ranged between 10 and 98 ms; mean ± SD: 42 ± 35 ms). Similarly, we clamped *G_0_* = 1.0 at 0 Hz. The remaining two parameters of the model that were free to vary across trials, namely the damping ratio and the system’s time constant, were found by MATLAB’s *procest* routine (process estimation). The amount of “damping” of the model is usually quantified by its so-called quality factor:

(5) $$Q_{n}\equiv \frac{1}{2\zeta_{n}}.$$

The impulse response in the time domain of the model is given by the following:

(6) $$h_{APS,n}\left(t\right)=L^{-1}\left[\exp(-T_{D}\cdot s\right]*L^{-1}\left[\frac{\omega_{C,n}^{2}}{\omega_{C,n}^{2}+2\zeta_{n}\omega_{C,n}s+s^{2}}\right],$$

(with * signifying convolution in the time domain). Noting that

$$L^{-1}[\exp(-as)]=\delta (t-a)\;andL^{-1}\left[\frac{b}{{\left(s+c\right)}^{2}+b^{2}}\right]=\exp(-ct)\cdot \sin\left(bt\right)\cdot u\left(t\right),$$

with *u(t*)=1 for *t *≥* *0 the Heaviside unit-step function, Equation 6 yields:

(7a) $$h_{APS,n}\left(t\right)=T_{C,n}\beta_{n}\cdot \text{exp}(-\omega_{C,n}\cdot \zeta_{n}(t-T_{D})\cdot \text{sin}\left(\omega_{C,n}\beta_{n}\cdot \left(t-T_{D}\right)\right)\;for\;\zeta_{n}<1,$$

(7b) $$h_{APS,n}\left(t\right)=T_{C,n}\gamma_{n}\cdot \left[\exp\left(-\omega_{C,n}\left(\zeta_{n}-\gamma_{n}\right)(t-T_{D})\right)-\exp\left(-\omega_{C,n}\left(\zeta_{n}+\gamma_{n}\right)(t-T_{D})\right)\right]\;for\;\zeta_{n}>1,$$

where $\beta_{n}\equiv \sqrt{1-\zeta_{n}^{2}},\gamma_{n}\equiv \sqrt{\zeta_{n}^{2}-1}$ and *t ≥ T_D_*.

To test how well this simple linear model accounts for the response data, we calculated the predicted head-position responses, *H_pred_(t)*, of the model for each subject and trial, *n*, by convolving the measured stimulus movement, *m_n_(t)*, with the fitted impulse response function of Equation 7 by the following:

(8) $$H_{Pred,n}\left(t\right)=\overset{\infty }{\underset{0}{\int }}m_{n}\left(t-\tau \right)\cdot h_{APS,n}\left(\tau \right)\cdot d\tau \;for\;n=1-30.$$

The gain characteristic of the underdamped response of the system (Q_n_ > 0.5, or 0 < ζ_n_ < 1) reaches its maximum value at frequency:

(9) $$\omega_{max,n}=\omega_{C,n}\cdot \sqrt{1-\frac{1}{4Q_{n}^{2}}}\;\;with\;G_{max}(\omega_{max,n})=\frac{4Q_{n}^{2}}{\sqrt{16Q_{n}^{2}-3}}.$$

The location of the peak approaches *ω_C,n_* for large *Q_n_* (with amplitude *Q_n_*). In case that *ω_C,n_*_=_ *ω_C_* (constant, as is found in our data), *ω_max,n_* decreases with decreasing Q*_n_*, together with the system’s maximum gain.

At Q_n_ = 0.5 the maximum gain (1.0) is reached at ω*_(max,n)_* = 0 (critically damped). Thus, the system’s effective time constant increases with decreasing Q_n_, while its overshoots decrease in size.

### Statistics

To quantify and evaluate how well the model represented the subjects’ responses, we calculated Pearson’s linear correlation coefficient, *r*, between the measured and predicted head-position time traces. We also determined the coefficient of determination, *r*^2^, which quantifies the variability accounted for by the model. In addition, we calculated the MSE of the model for each trial. The effect sizes and confidence intervals (CIs) are reported as effect size (CI width lower bound; upper bound).

We also estimated the mean perceived absolute pursuit error (in degrees) during each trial, *n*, for each subject, s, by calculating:

(10) $$MAE_{n,s}=\frac{1}{20}\overset{20}{\underset{0}{\int }}\left|m_{n}\left(t\right)-H_{n,s}\left(t+T_{Opt,n,s}\right)\right|\cdot dt,$$

with $H_{n,s}\left(t+T_{Opt,n,s}\right)$ the measured head movement of subject *s* in trial *n*, leading by *T_Opt,n,s_* ms with respect to the stimulus movement, *m_n_(t)*. This delay was found by a brute-force search for the value that would minimize the mean absolute error (Eq. 10) between target and head movement during that trial (see Figs. 7, 8*A*,*B*).

**Table 1:** Linear regression on instantaneous head position (H) as function of the head-position error (ΔE; **Eq. 11b), and on head velocity (**$\dot{H}$**) as function of the velocity error (**$\Delta \dot{E}$**; Eq. 11a)**

Regression	Offset	Slope	Correlation
H=α+β·ΔE	−0.98	−1.13	0.55
$\dot{H}=\rho +\eta \cdot \Delta \dot{E}$	0.00	−0.96	0.63
$H=a+b\cdot \Delta \dot{E}$	0.16	−0.009	0.01
$\dot{H}=c+d\cdot \Delta E$	0.20	0.15	0.03

Both regressions yield high gains and correlations. Head position is unrelated to the velocity error, and, conversely, head velocity is independent of the position error. All regressions were performed on a subset of 48,400 data points (trajectories were sampled in steps of 15 samples, because of memory limitations).

To quantify a potential change in the model parameters across trials, we fitted a hierarchical linear regression model, obtaining slopes and intercepts for each subject and for the group as a whole (Kruschke, 2014) via the sampling program JAGS through MATLAB (Plummer, 2003; Steyvers, 2011). We report the mean and 95% highest-density intervals (HDIs) for the slopes of the fitted lines.

## Results

### Example auditory pursuit

**Figure 3.**
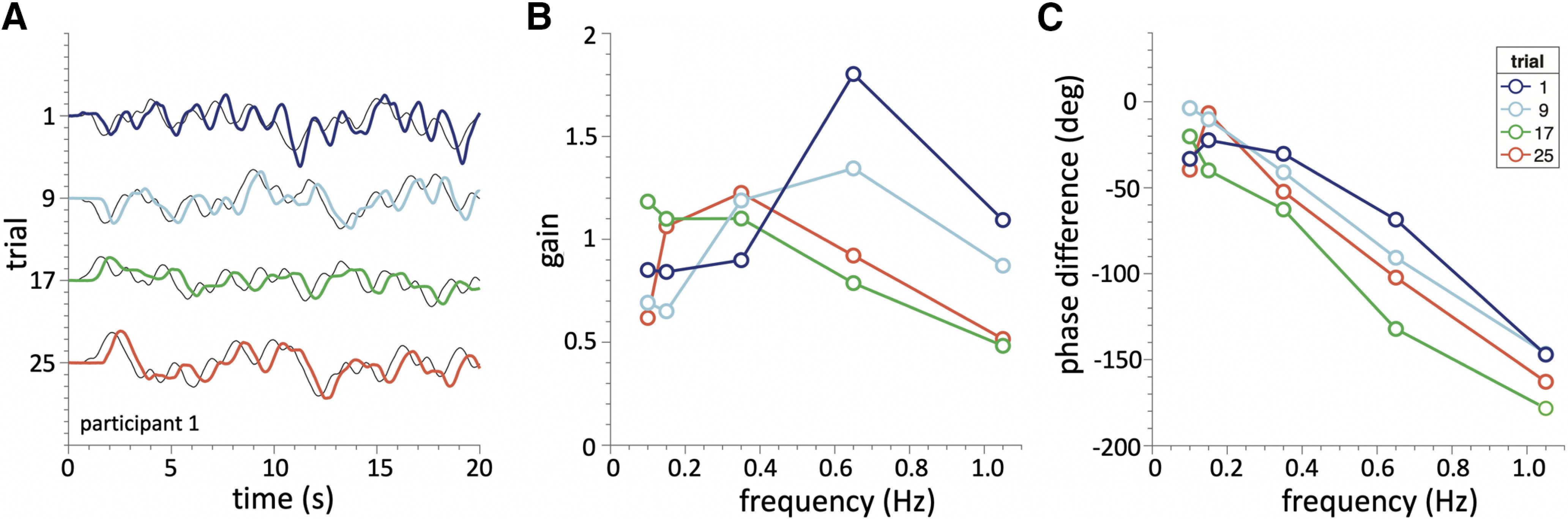
Examples of auditory pursuit to unpredictable sound movements. Four representative trials from subject S1. ***A***, Thin black traces: stimulus movement; colored traces: head movements. The stimulus movement contained five discrete harmonics with random phases (Eqs. 1, 2). ***B***, ***C***, Transfer characteristics (gain, and phase, in degrees; linear scale) of the stimulus-response relations at the five stimulus frequencies (open dots) for each trial. Both characteristics vary with trial number.

We first illustrate the pursuit behavior of our subjects by showing some representative stimulus-response traces to four unpredictable sound-source movements and their associated transfer characteristics for subject S1 (Fig. 3). A qualitative inspection of the traces (Fig. 3*A*) indicates that the subject’s head movements lagged the stimulus movements during the entire trial, with an average delay of ∼300 ms. Leading movements were not observed in these trials. The smooth-pursuit head movements were in the direction of stimulus motion, and corrective fast head saccades were not detected at any stage of the stimulus presentation. To determine the transfer gain and phase characteristics for the five major frequencies in the motor movements (Fig. 3*B*,*C*), we applied the fast Fourier transform to the stimulus and response traces. The subject’s gains (response amplitude divided by stimulus amplitude at each stimulus frequency) tended to fall off at higher frequencies, with its highest value at intermediate frequencies, suggesting a bandpass response behavior. A qualitative inspection of these gain and phase characteristics suggests that they systematically change with trial number.

### APS identification

**Figure 4.**
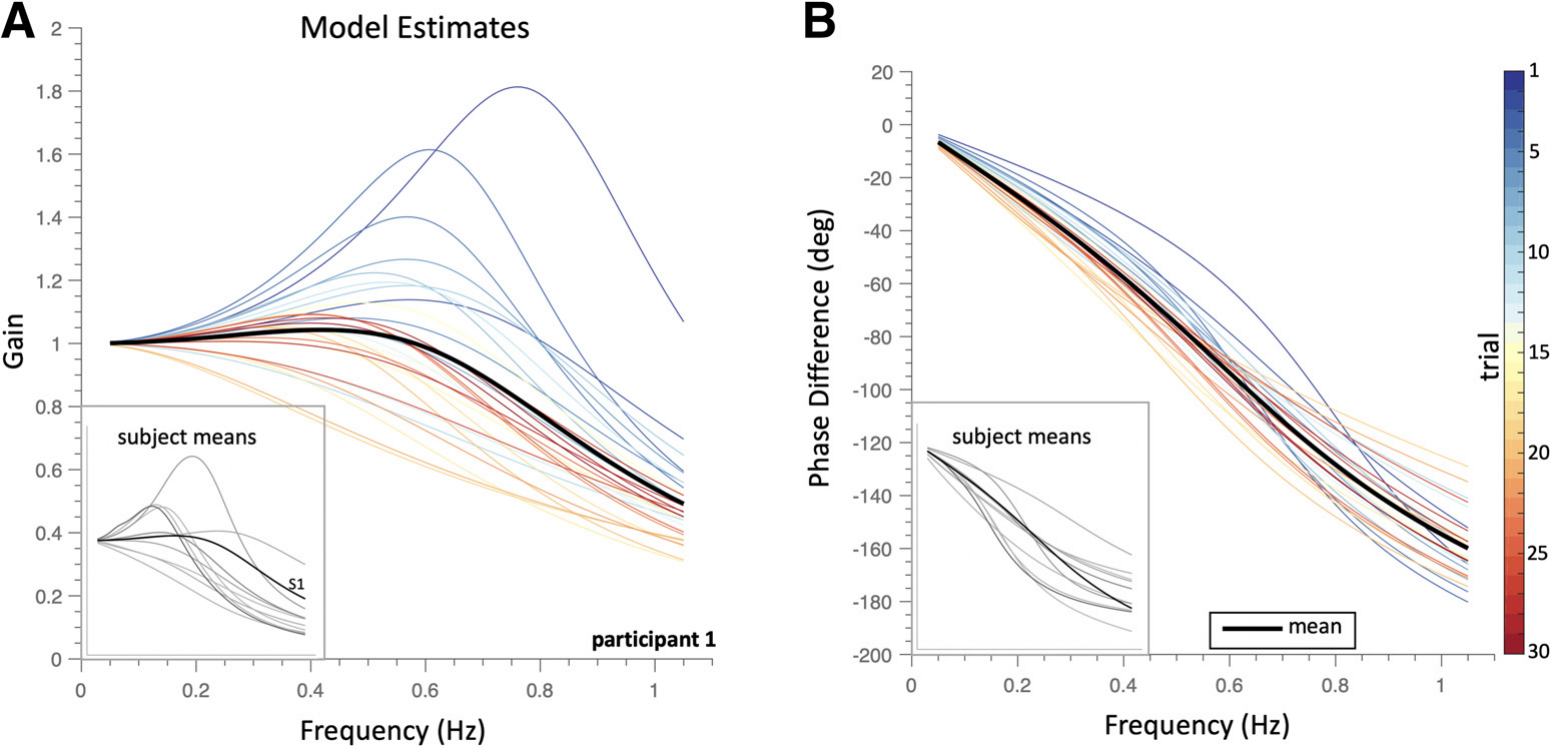
Estimated model fits for participant S1 (Eq. 4). Gain-characteristics (***A***) and phase-characteristics (***B***) for the stimulus-response data of all 30 trials as function of frequency. The thin curves show the results for each trial, color coded by trial number. The black solid lines correspond to the average transfer characteristic across trials. Note the systematic change of the gain and phase characteristics with trial number. Insets, average gain and phase characteristics for all 11 subjects.

Based on the qualitative observations in Figure 3, we modelled the system’s responses in each trial by the second-order filter characteristic of Equation 4. Figure 4 illustrates the individual fitted transfer functions from subject S1 for all 30 trials (thin colored lines), together with the averaged gain (dimensionless; Fig. 4*A*) and phase characteristics (in degrees; Fig. 4*B*, bold black lines).

The results suggest a systematic change of the model parameters with trial number: the size and location of the peak of the amplitude characteristic gradually shifted from higher to lower frequencies with trial number (Fig. 4*A*). Similarly, the phase characteristics changed gradually with trial number from higher to lower frequencies at each given lag. We obtained similar results for the other participants (Fig. 4, insets).

**Figure 5.**
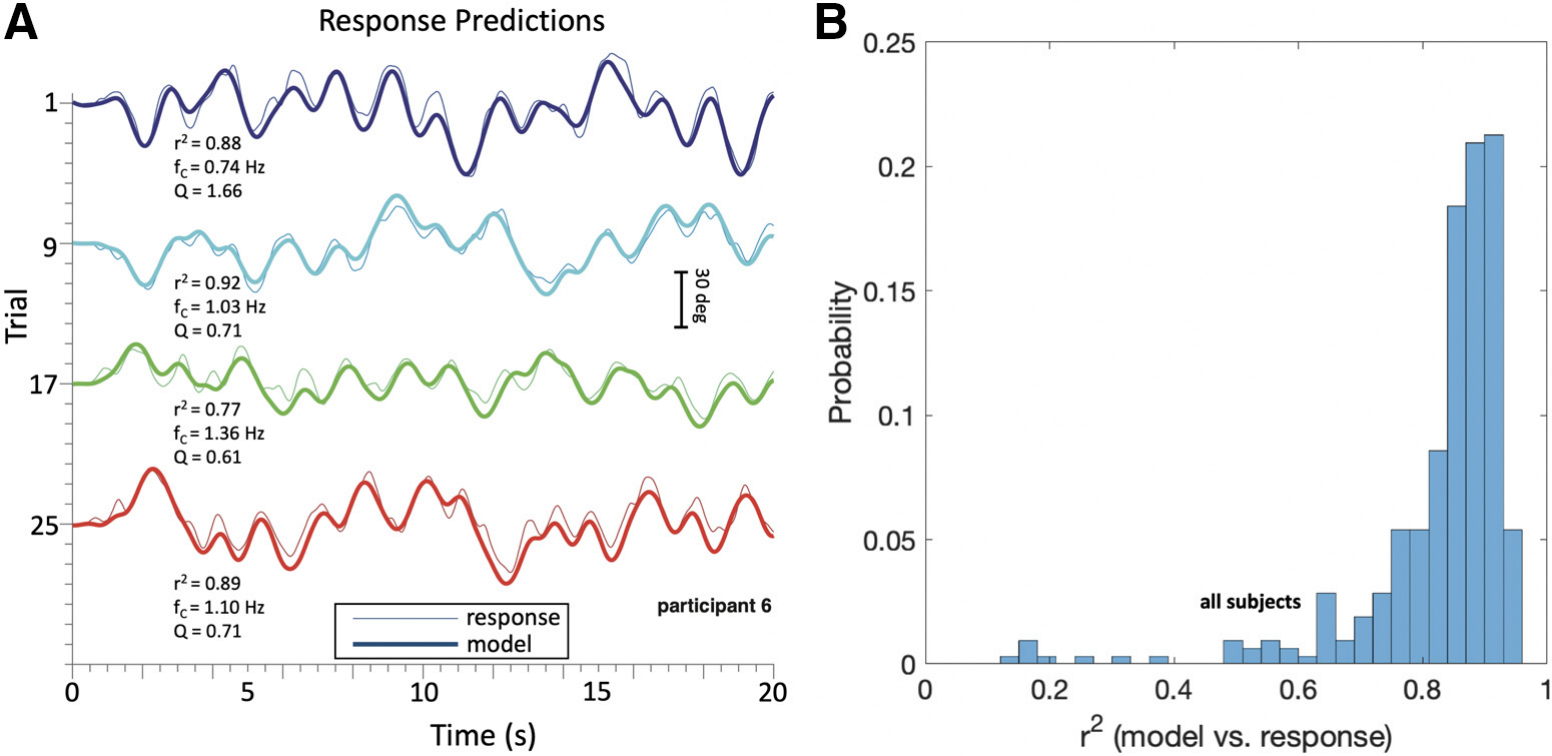
***A***, Measured (thin traces) and predicted (thick traces; Eq. 8) head-movement responses for four representative trials of subject S6. Each trace shows head orientation as a function of time. Optimal fit parameters (f_C_, Q, and goodness of fit, *r*^2^) are provided for each trial. Note the good correspondence between measured and predicted movements. The subject’s response delay was clamped at T_D_ = 98 ms for all trials. ***B***, Histogram of the coefficients of determination, *r*^2^, for all 315 trials (11 subjects). The mode of the distribution lies at *r*^2^ = 0.88, which indicates an excellent fit of the data (variance explained).

To test whether the simple feedforward second-order filter was able to account for the full stimulus-response behavior of the participants, we calculated the predicted responses through convolution (Eq. 8) of the model’s impulse response function for each trial, *h_APS,n_(t)* (Eq. 7), and the measured stimulus movement, *m_n_(t)* (Eq. 2). Figure 5*A* shows four representative examples of the measured (thin colored traces) and predicted responses (bold colored traces) of participant S6. Figure 5*B* provides the coefficients of determination (*r*^2^) for all 11 subjects and trials. These results show that the simple model of Equations 4, 7 provided an excellent description of the response data for all participants and the far majority of trials, with the mode of the distribution at *r*^2^ = 0.88 (i.e., 88% of variance explained; across subjects: mean ± SD: 0.83 ± 0.10).

### Adaptive changes in auditory pursuit

**Figure 6.**
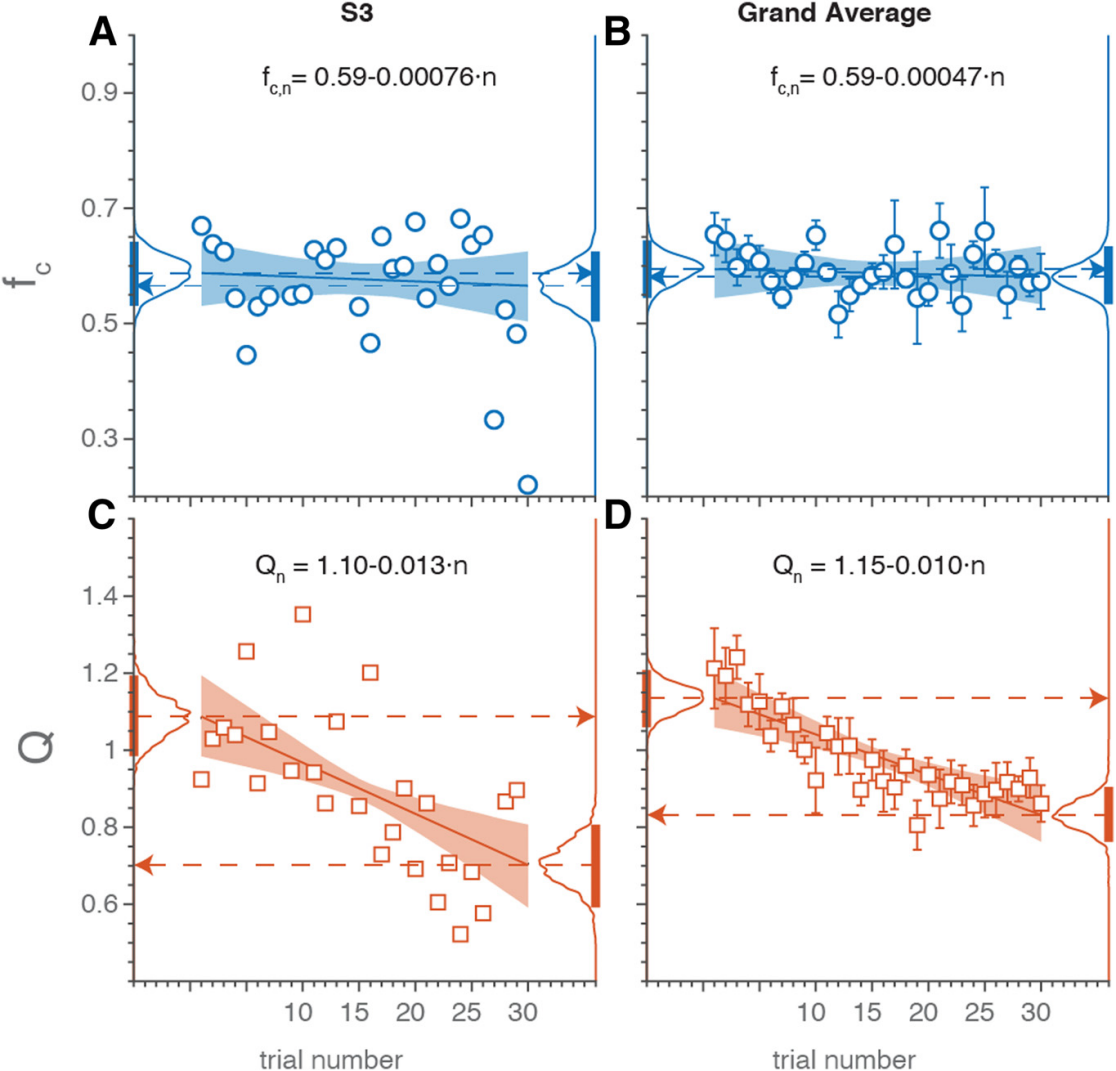
Adaptation of auditory pursuit. ***A***, ***B***, The model’s resonance frequency, f_C_ and quality factor, Q (***C***, ***D***), as a function of trial number for subject S3 (***A***, ***C***), and pooled across all subjects (***B***, ***D***). Shaded areas indicate the 95%HDI for the regression lines through the data. For the pooled results, a hierarchical regression analysis was performed, which accounts for the individual differences. Histograms on the left and right of each panel show the distributions of the respective parameter at the start (trial 1), and end of the experiment (trial 30), respectively, with their HDI (bars). The system’s resonance frequency did not change systematically with trial number, while the quality factor decreased systematically, and highly significantly, throughout the experiment. The equations in each panel denote the optimal regression results.

Figure 4 suggests a systematic change of the model characteristic as a function of trial number. To quantify this trend in the pursuit behavior of all subjects, we performed a linear regression analysis (for details, see Materials and Methods) on the two free parameters of the model: its center frequency, *f_C,n_*, and the quality factor, *Q_n_*, as a function of trial number, *n* (Fig. 6).

We first illustrate the results for subject S3 in Figure 6*A*,*C*. The center frequency (Fig. 6*A*, blue) did not change systematically during the experiment, with the data (open circles) scattering around the average value of *f_C_* ≈ 0.6 Hz. The optimal regression line through the data (solid line) had a slope close to zero (slope = −0.8 × 10^−3^; 95%HDI = [−4.4,+2.8] × 10^−3^. The *f_C_* values predicted by the linear model at the start of the experiment (at trial 1, inset histogram and 95%HDI bar on the left) and at the end of the experiment (trial 30, inset histogram and bar on the right) were also similar (the probability densities indicated by the histograms and bars for trial 1 and 30 overlapped considerably).

This subject’s quality factor (Fig. 6*C*, open squares) seemed to decrease on average although variability between individual trials was considerable. Nevertheless, the optimal slope was clearly non-zero (−13 × 10^−3^; 95%HDI = [−21,−7] × 10^−3^) and the variability in the fitted lines was low (as reflected by the shaded area indicating the 95%HDI of the regression). Similarly, the most likely Q- values at the first and last trials did not overlap at all (compare left and right histograms and bars).

Very similar results held for all 11 subjects (Fig. 6*B*,*D*). The center-frequency data averaged across subjects (Fig. 6*B*, open circles) scattered around a value of *f_C_* ≈ 0.6 Hz, and did not vary significantly during the experiment. The slope of the optimal average regression line was near-zero (−0.5 × 10^−3^; 95%HDI = [−3.5,+2.8] × 10^−3^). In contrast, the average quality factor (Fig. 6*D*, open squares) decreased substantially as the experiment progressed, from values higher than 1.0 in the early phase of the experiment (indicating an under-damped, bandpass behavior) toward the end of the session. This change was also reflected in the group regression slope of −10 × 10^−3^ (95%HDI = [−15,−5.5] × 10^−3^). Overall, the predicted total change of the quality factor across the 30 trials was −0.30, a nearly 27% difference.

**Figure 7.**
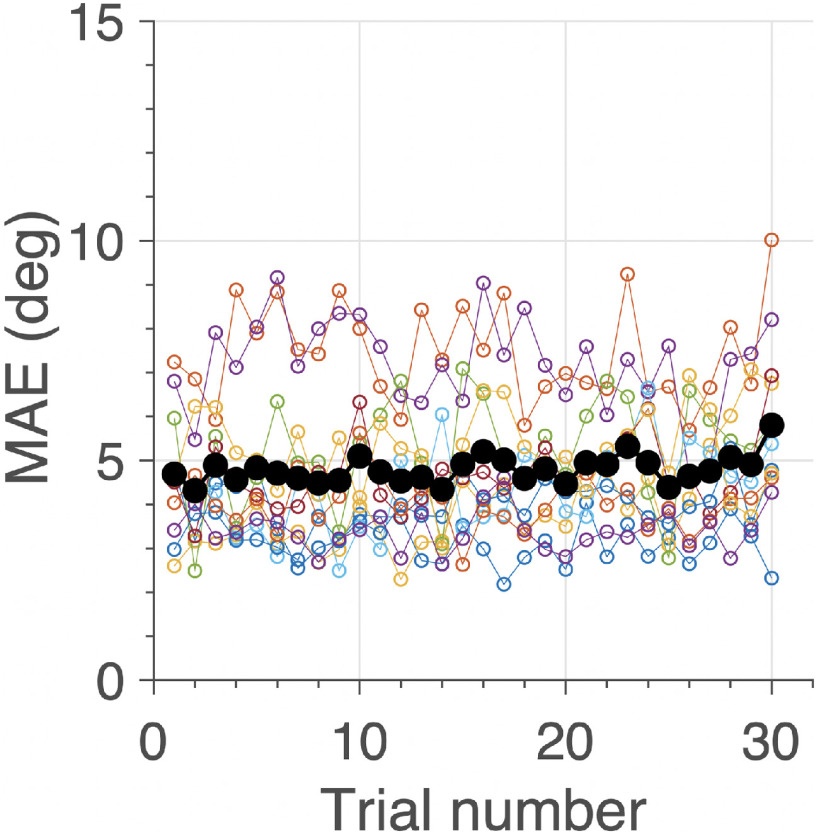
MAE (in degrees; Eq. 10) as function of trial number for each subject (different colors), and the grand averages across subjects (solid black dots). There was no trend for a change (neither positive, nor negative) in the MAE with trial number.

To test whether the change in the response characteristics would lead to improved pursuit performance we calculated the mean absolute localization error (in degrees) for each trial (Eq. 10). Figure 7 shows the results of this analysis for each participant (color coded) and for the average behavior across subjects (black dots). The data show that the mean absolute error across participants remained constant at 4.8° (SD 1.7°) throughout the experiment.

### Position error versus velocity error

The head-movement responses of our subjects contained the same spectral motion components as the stimulus, which suggests that the responses may have been driven by sound-source velocity. However, Rashbass (1961) noted for visual pursuit that there is a theoretical possibility that the pursuit system samples target positions at a sufficiently high rate that exceeds the spectral bandwidth of the response system. In that case, discrete position sampling is indistinguishable from smooth pursuit of target velocity. To rule out the former would require a paradigm in which a directional change in position error is dissociated from the direction of the smooth target movement. To our knowledge, such an experiment has not yet been conducted for auditory pursuit.

However, the random stimulus movements in the current experiment might in principle allow for some dissociation between these two variables. To check for this possibility, we performed a trial-by-trial regression analysis on the instantaneous head velocity versus head-velocity error, and on the current head position versus head-position error, respectively:

(11a) $${\dot{H}}_{n,s}\left(t+T_{Opt,n,s}\right)=\alpha +\beta \cdot \Delta {\dot{E}}_{n,s}\left(t\right),$$

(11b) $$H_{n,s}\left(t+T_{Opt,n,s}\right)=\varrho +\eta \cdot \Delta E_{n,s}\left(t\right),$$

where position error is defined as $\Delta E_{n,s}\left(t\right)=m_{n}\left(t\right)-H_{n,s}\left(t+T_{Opt,n,s}\right)$ and head-velocity error by $\Delta {\dot{E}}_{n,s}\left(t\right)={\dot{m}}_{n}\left(t\right)-{\dot{H}}_{n,s}\left(t+T_{Opt,n,s}\right)$. Here, α, β, ρ, and η are the regression parameters obtained for the entire data set, and *m_n_(t)* is the sound-movement trajectory of trial *n*. *T_Opt,n,s_* is the optimal delay of the head movement found in trial *n* for subject *s*.

**Figure 8.**
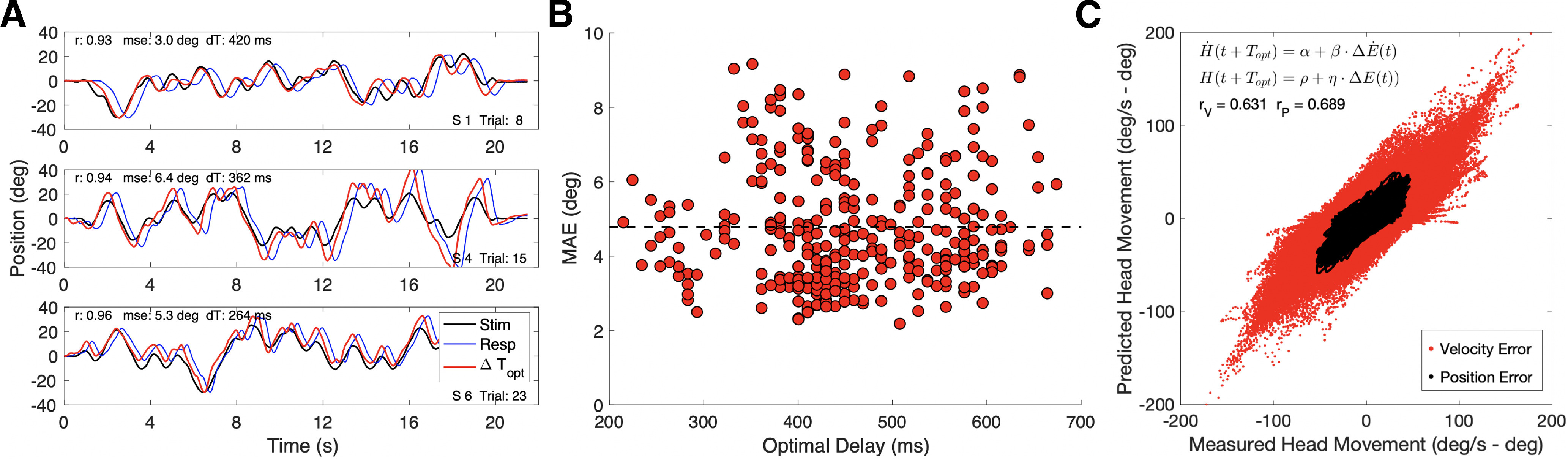
***A***, Three example trials for three different subjects, showing the stimulus movement (black), the head movement (blue), and the time-shifted head movement that aligns best with the stimulus (red). *r*: correlations between stimulus and time-shifted head-movement traces are very high. MSE: the mean-absolute errors in degrees. dT: the applied optimal shift (in milliseconds). ***B***, Distribution of the mean absolute errors and optimal delays for all trials and subjects (*N* = 330). ***C***, Measured instantaneous (optimally shifted) head velocity versus the predicted head velocity (Eq. 11b; red) and measured head position versus predicted head position (Eq. 11a; black). Both models correlate well (*r*_V_ = 0.63 vs *r*_P_ = 0.69; total number of data points: *N* = 726,000; see also Table 1). The errors themselves are uncorrelated (*r* = −0.01).

Figure 8 presents the results of this analysis. Figure 8*A*,*B* illustrates the procedure of finding the optimal time-shift of the head trajectory, *T_Opt,n,s_*_,_ such that it aligned best with the stimulus trajectory during the trial [yielding the smallest mean absolute pursuit error (MAE)], for three different subjects and trials. Note the very high correlations between stimulus movement and time-shifted head movement (*r* > 0.9, for 2200 data points). Figure 8*B* shows the joint distribution for all trials and subjects of best delays and minimum MAEs (the latter also shown in Fig. 7*A*). Figure 8*C* shows the predictions of Equations 11a (red) and 11b (black) for the entire dataset (11 subjects × 30 trials × 2200 samples = 726,000 points). Both regressions yield a high correlation, indicating that subjects followed the pseudo-random stimulus trajectories quite faithfully (Table 1). Note the slightly higher correlation for the position error predictor (*r*_P_ = 0.69) than for the velocity error predictor (*r*_V_ = 0.63), while the two errors themselves were uncorrelated (*r* = −0.01). Moreover, the head velocity was unrelated to the instantaneous sound position (*r* < 0.001; see Table 1). Thus, the head-velocity error and head-position error both described the head-movement data (velocity and position) about equally well.

## Discussion

Our results show that subjects smoothly track broadband sounds, moving along unpredictable trajectories in the horizontal plane, with remarkable accuracy. The frequency spectrum of their head-movements contained the same dominant frequencies as the source-movements. We described the tracking responses by a simple feedforward second-order linear filter and found that its damping gradually increased during the experimental session. A slightly underdamped bandpass response in early trials, turned into an overdamped response with a longer effective time constant (Eq. 9) and lag (Figs. 4, 6). We argue that this behavior reflects an adaptation of the APS to a learned stimulus characteristic. In what follows, we discuss these features in more detail.

### Pursuit accuracy

Although ocular pursuit of visual targets is well documented and modelled (Robinson et al., 1986; Krauzlis and Lisberger, 1994; Barnes, 2008), much less is known about the cranial pursuit of moving sounds. Earlier eye-movement studies have reported poor auditory pursuit performance (gain < 0.2; Boucher et al., 2004; Berryhill et al., 2006) and concluded that the auditory system has no specialized motion detectors, because of its coarse spatial acuity compared with vision. However, we argue that lack of accurate eye-movement tracking to moving sounds is not evidence for absence of motion-sensitive auditory processing.

The ocular pursuit system successfully tracks visual targets to reduce retinal slip-velocity through continuous visual feedback (Robinson et al., 1986; Krauzlis and Lisberger, 1994; Lisberger, 2010). While visual-cortical and subcortical areas contain pursuit-sensitive and target-velocity sensitive neurons (Mikami et al., 1986; Dürsteler et al., 1987; Ilg and Thier, 2008), evidence for auditory motion-sensitive cells has been obtained for sensory responses under anaesthetized conditions only (see Introduction). Importantly, however, head-fixed ocular pursuit of sounds does not reduce any error-signal, as the head-centered acoustic information does not change with eye movements. Therefore, it is questionable whether ocular pursuit of sounds may serve as a valid measure for sound-velocity processing. Instead, cranial pursuit does affect the sound’s acoustic cues, in a way that is directly related to self-initiated head movements (Fig. 1).

We here demonstrated that subjects successfully tracked unpredictably moving sounds with smooth head-movements that were always in the stimulus direction at a fixed, idiosyncratic delay (Figs. 3, 8*A*). Subjects could not anticipate the pseudorandom changes of the target’s movement direction as indicated by a constant mean absolute error across trials (Eq. 10) of ∼4.8° (Fig. 7). This indicates that the system did not attempt to reduce the perceived pursuit error, presumably because it could not rely on any predictions for these random trajectories. As a result, the head-movement delay during a trial amounted to several hundreds of milliseconds (Fig. 8*A*,*B*), which did not change systematically across trials, and was unrelated to the mean absolute error in a trial (Fig. 8*B*).

Sound-localization experiments with eye-head movements have demonstrated that the auditory system continuously uses eye-movement and head-movement information to update the location of brief sounds (Vliegen et al., 2004; Genzel et al., 2018). We have hypothesized that the APS aims to keep its AF close to the target, just as in visual pursuit (Fig. 1). A putative AF would, by definition, correspond to the region of highest spatial acoustic resolution. For ITDs and ILDs, which vary sinusoidally with the azimuth angle (Blauert, 1997; Van Opstal, 2016), the AF would be around straight-ahead, with 1.0–1.5° acuity (Mills, 1958). Interestingly, an acoustic spatial fovea is not anatomically represented in the cochlea. It is therefore an abstract, functionally defined concept, neurally generated from binaural integration of different acoustic processing streams. Although it is not yet known how the brain represents an AF, or what the relative contributions of the ITD and ILD pathways are, our data may support its functional existence (see also below).

### Adaptive response behavior

The adaptation of pursuit-responses across trials gradually increased the system’s damping. The other parameters of our model (time constant, *T_c_*, and processing delay, *T_D_*) did not change systematically with trial number (Fig. 6). It is not immediately obvious which cost the pursuit system aimed to optimize, as multiple factors may underlie the cost evaluation: position and velocity errors (response accuracy), movement effort (energy consumption), response duration to match target velocity (discount of reward), trajectory smoothness, etc. As experiments were performed under open-loop conditions, participants never obtained exogenous feedback about the true target trajectory, and had to rely entirely on ongoing endogenous processing of acoustic information, together with self-initiated head-movements, and associated vestibular and efference-copy signals. This situation differs radically from classical visual pursuit.

Target-movement trajectories were unpredictable from trial to trial, and also within a trial. Thus, a possible pursuit strategy could have been to generate head-movements through a fixed input-output characteristic. Our data show that in the first couple of trials this characteristic could be well described by a slightly underdamped impulse response (Eq. 7), for which the frequency-characteristic has maximum gain (>1.0) around a cutoff frequency of 0.6–0.8 Hz. Interestingly, visual pursuit to an unexpected change of target velocity is characterized by a similar “ringing” of pursuit eye-velocity at 3–3.5 Hz (Robinson et al., 1986; Krauzlis and Lisberger, 1994).

Remarkably, the APS seemed to extract implicit spectral information from evoked movement trajectories in the stimulus ensemble, and gradually changed its response behavior, such that the underdamped characteristic became near-critically damped, with Q ∼0.75 (Q = 0.5 is the critically-damped response). To verify that this is indeed the case, future experiments could manipulate the amount of consistent spectral information in the movement trajectories, and test whether it affects the long-term pursuit behavior.

### Response strategy

**Figure 9.**
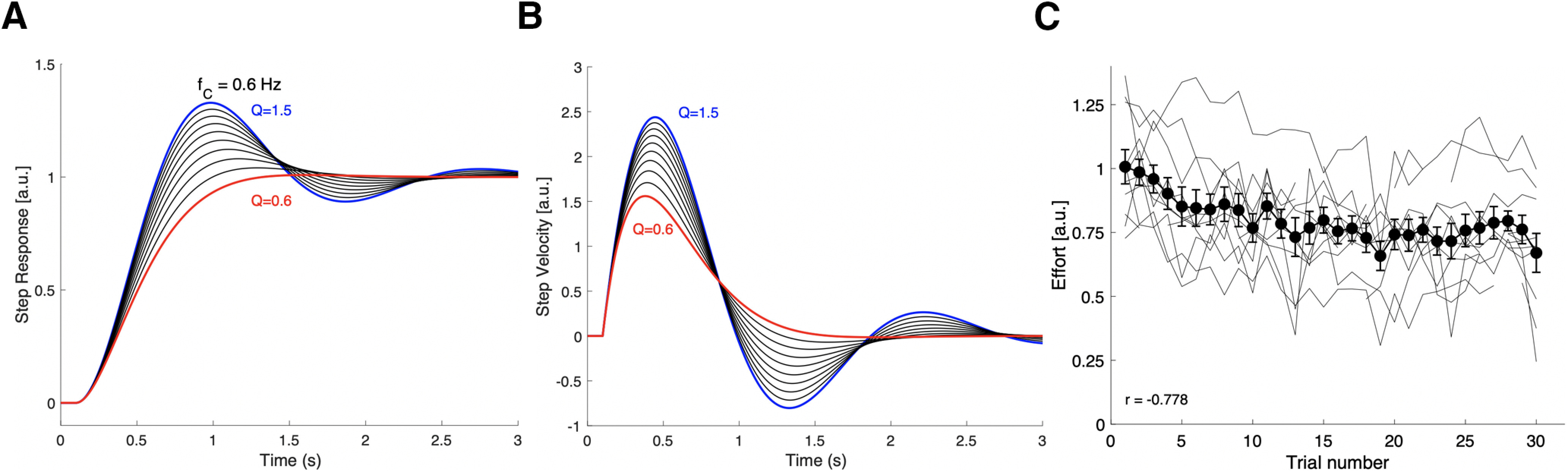
***A***, The model’s unit-step responses for 10 values of Q between Q = 1.5 (blue) and 0.6 (red) in decreasing steps of −0.1 (black). ***B***, The system’s response velocity (i.e., its impulse response) for the unit-step input to the same Q values. At Q = 0.6, the system is near-critically damped and reaches its equilibrium value much faster than for higher Q values. In addition, the total consumed energy by the system is considerably less at the lower Q: from Q = 1.5 to Q = 0.6 the total cumulative energy reduction is 60%. ***C***, Calculated effort from the fitted gain characteristics of Equation 4, taken as the mean spectral power over 0.05–1.05 Hz for all 11 subjects (thin gray lines), and the mean across subjects (black solid line), plotted as function of trial number. The correlation for the mean is *r* = −0.778; it decreases by 34% between the first and the last trial.

We propose that for the pseudo-random stimulus set this adaptive response strategy may have optimized a cost that included the system’s response-duration and total response effort. Simple estimates of these costs can be made from the model’s response characteristic. Figure 9*A* illustrates the step responses, and Figure 9*B* the associated impulse responses, of the second-order model of Equation 7, for which we took a fixed resonance frequency of *f_C_* = 0.6 Hz, and the quality factors varying between Q = 1.5 and Q = 0.6 in steps of −0.1, as obtained in our experiments (Fig. 6).

The duration of the step-response is clearly shortest for the lowest Q factor (D_min_ ∼ 1.8 s). If we assume that the total (rotational kinetic) energy consumption during the head movement is proportional to its absolute squared (angular) velocity, or to the mean spectral power of the system’s amplitude characteristic, per unit of angular momentum:

(12a) $$E_{Q}(D)=\left|\overset{D}{\underset{0}{\int }}v^{2}\left(t\right)dt\right|$$

or

(12b) $${\bar{E}}_{Q}=\frac{1}{\left(\omega_{max}-\omega_{min}\right)}\cdot \overset{\omega_{max}}{\underset{\omega_{min}}{\int }}\left|G(\omega )\right|d\omega ,$$

then also E_Q_ reaches a clear minimum for Q = 0.6 (Fig. 9*B*). This also holds when the integration window is kept fixed at the minimum D = 1.8 s for all Q values, e.g., for Q = 1.5, E_1.5_(1.8) = 2.79, and for Q = 0.6: E_0_._6_(1.8) = 1.13, which is a reduction of 60%.

In Figure 9*C*, we plotted the estimated mean absolute spectral power (in arbitrary units) of the fitted gain characteristic over 0.05–1.05 Hz for each trial of all 11 subjects, as a function of trial number. The results show that the total effort estimate indeed decreased systematically during the course of the experiment, by ∼34% (difference between the first and last trial), with an overall correlation of *r* = −0.78.

Minimization of an overall performance cost has also been suggested by others to underlie oculomotor behavior (for eye saccades, Harris and Wolpert, 2006; Sadaghat-Nejad et al., 2019). As the human fovea has a high resolution within only 1° of visual angle, and considerable uncertainty in the retinal periphery, theoretical studies have indicated that the saccadic system aims to optimize speed-accuracy trade-off, to minimize saccade duration at the smallest mean-absolute localization errors (Harris and Wolpert, 2006).

Similar optimization principles appear to hold for human sound localization (Ege et al., 2019). Note that despite the availability of acoustic cues for azimuth and elevation, veridical localization of a sound is not possible with these cues alone, as the sensory spectrum results from a convolution of source spectrum and pinna cues, both of which are a priori unknown. Thus, the brain cannot be sure about the veridical source direction without making prior assumptions (Middlebrooks and Green, 1991; Van Opstal, 2016). Experiments have suggested that the auditory system uses several priors, learned through experience: for example, (1) each pinna filter refers to a unique elevation angle, and (2) natural source spectra do not resemble the pinna spectra (Hofman and Van Opstal, 1998), (3) not all spectral bands of the pinna filters are equally informative (Zonooz et al., 2019), and (4) not all source locations are equally likely (Ege et al., 2018, 2019). We recently demonstrated that the auditory system reweighs its spectral and source-location priors within the same experimental session, without exogenous visual feedback (Zonooz et al., 2018; Ege et al., 2019), suggesting that the brain combines the acoustic input across trials, in combination with its own head-orienting commands, to update its priors. Our results provide evidence for a similar strategy when tracking moving sounds.

### Neural implications

The success of the simple linear feedforward model in predicting auditory pursuit in azimuth does not exclude the possibility that the system may actually be driven by dynamic feedback, in which the neural estimates of craniocentric source velocity and source position result from the combined effect of the true target velocity and position with the self-generated signals related to head-velocity and change in head position, like suggested in Figure 1. A pursuit system would aim to minimize the estimated auditory foveal slip-error, by ensuring that the instantaneous target estimate remains close to the representation of an AF. For that, it should ensure that both the head velocity should be similar to target velocity, and that the AF should be close to the target position. The analysis shown in Figure 8*C* suggests that the craniocentric velocity and position errors both contribute strongly to the head-pursuit behavior.

**Figure 10.**
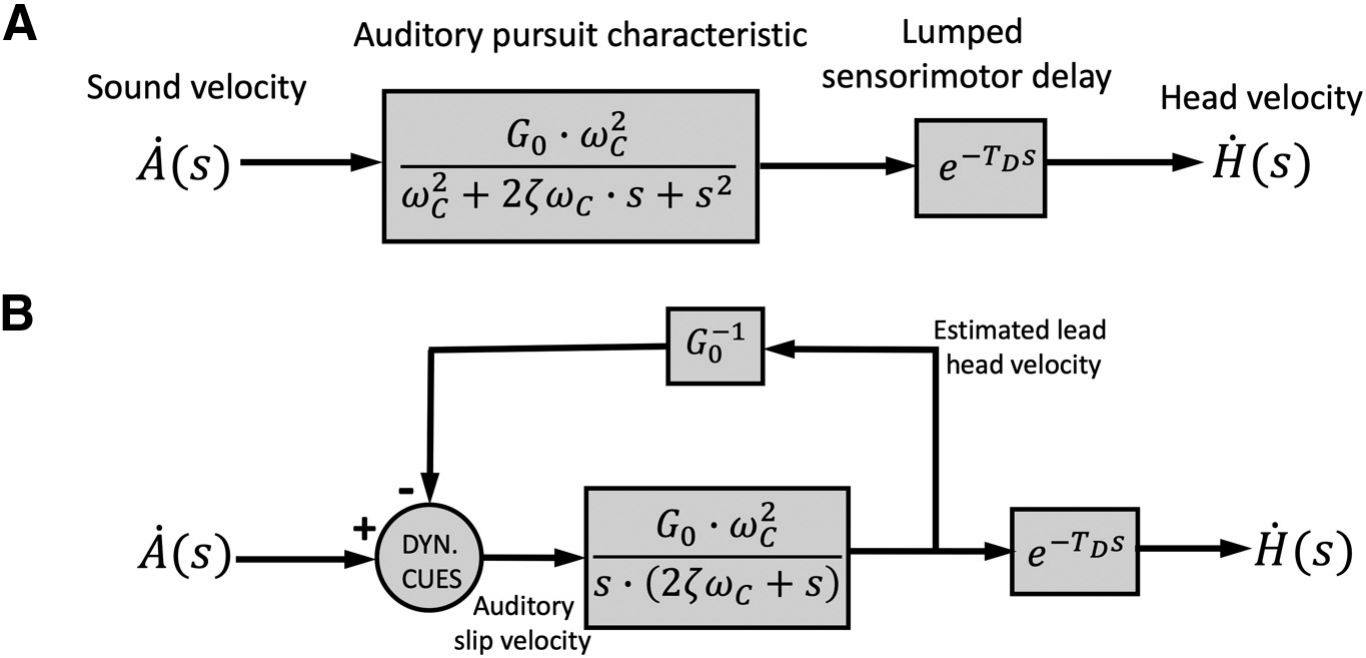
Models. Two mathematically equivalent schemes for the APS, on the basis of our results. Both models are represented in the Laplace domain. ***A***, Feedforward implementation of Equation 4: a second-order low-pass filter in series with a lumped sensory-motor delay. ***B***, Equivalent feedback model, in line with the proposal in Figure 1; the feedback path carries an internal estimate of the output head velocity with a scalar gain, 1/G_0_, and lead, T_D_. The feedforward path has a pole in zero (pure integrator), and at s = −2ζω_C_ (i.e., a leaky integrator with time constant, T_FF_ = (2ζω_C_)^−1^. The feedback comparator computes the auditory slip velocity, which in this model would be given by $v_{SLIP,C}\left(t\right)=\dot{A}\left(t\right)-G_{0}^{-1}\cdot \dot{H}\left(t+T_{D}\right)$. For simplicity, the head-position error is not included in this scheme.

A putative example of a feedback system, incorporating our results, is illustrated in Figure 10*B*.

Note that in visual pursuit, feedback is automatically implemented, as retinal slip is locked to the moving eye. This is less trivial for auditory pursuit, as an auditory (spatial) fovea is not linked to the basilar membrane; its representation should result from neuro-computational mechanisms. Figure 1 indicates the major neural pathways and computational stages for tracking moving sounds in azimuth. Moving sounds produce dynamic changes in the high-frequency ILDs and low-frequency ITDs, processed in binaural brainstem pathways that terminate in the Superior Olivary complex (LSO and MSO; Yin, 2002). Together, the outputs of these pathways converge on IC, a central hub for spatial and spectral-temporal processing of sounds (Groh et al., 2001; Casseday et al., 2002; Zwiers et al., 2004; Versnel et al., 2009). The IC would therefore be the prime target to study tuning to head-centered sound-source velocity and position error, for which some evidence has been obtained from dichotic experiments in anesthetized cats (Al’tman et al., 1985; Bekhterev, 2003), owls (Wagner and Takahashi, 1992), guinae pigs (Ingham et al., 2001), and bats (Pollack, 2012).

Further evidence from cats (Toronchuk et al., 1992), rats (Doan and Saunders, 2003), and humans (Kreitewolf et al., 2011) suggests that also auditory-cortical areas may be responsive to sound velocity. We conjecture that our results may hint at the interesting possibility that these cortical cells could instead encode auditory slip-error in velocity and position with respect to the AF (Figs. 1, 10*B*), in a similar way as ocular pursuit-responses in visual-cortical areas (Mikami et al., 1986; Dürsteler et al., 1987). To demonstrate this, however, will require electrophysiological recordings from behaving animals, trained to track moving sounds with the head, which have so far not been performed.

Indeed, as single-unit recordings have demonstrated clear behavioral correlates at different stages in the monkey auditory system (Groh et al., 2001; Zwiers et al., 2004; Massoudi et al., 2013, 2014), we propose that inclusion of the full action-perception cycle is essential to understand the neural processing of moving sounds (Van Opstal, 2016).

## Synthesis

Reviewing Editor: Leonard Maler, University of Ottawa Decisions are customarily a result of the Reviewing Editor and the peer reviewers coming together and discussing their recommendations until a consensus is reached. When revisions are invited, a fact-based synthesis statement explaining their decision and outlining what is needed to prepare a revision will be listed below. The following reviewer(s) agreed to reveal their identity: Stephen Lisberger, Dardo Ferreiro.

Editor comments

Both reviewers find much to like in this work and note that the study is well motivated and generally sound. Both reviewers also have concerns about specific aspects of the research. The concerns are not fatal and should not be difficult to address and both reviewers hope to see a revised manuscript.

Please make the revisions suggested in the detailed reviews. Be sure to summarize the changes you have made in a rebuttal and also provide a redlined version of the ms so that the reviewers can readily see how you have altered the Ms to meet their critical points.

Reviewer #1

This is a sound study that provides new data and an interesting insight into tracking of moving auditory stimuli. It has multiple strengths and two serious flaws, both fixable.

Strengths:

(1) the modeling framework seems sound and the data are new, clean, and properly analyzed; (2) the concept of an “auditory fovea” is new for me and it comes from the revelation (also new for me) that the head (rather than the eyes) should smoothly track moving auditory stimuli; (3) the adaptation of system performance across trials is interesting and makes sense.

Weaknesses:

The first flaw is the conclusion that the head tracks auditory target velocity and therefore that there must be an auditory motion system in the brain as there is for visual motion. But the study presents no evidence that the head is tracking motion. It could as easily be tracking auditory position and neither critical data nor critical analysis is presented to discriminate these two possibilities. For eye pursuit, it is the step-ramp of Rashbass (1961!) that proves the system is tracking motion, but that kind of experiment probably isn’t going to work for head tracking of auditory stimuli. I think the authors just need to back off here.

The second flaw is the structure of Figure 1 and this seems like a fundamental issue. It does not invalidate the study, but it fails to provide a proper conceptual framework and needs to be fixed. The auditory brainstem should be sensing sound velocity relative to the (moving) head, which will be auditory slip velocity by the authors’ terminology. I don’t see exactly how to fix this other than to feedback head velocity all the way to the beginning of the diagram. Also, I think that the authors are trying to make an analogy to the Robinson view of the visual smooth pursuit system where he proposed that “target velocity in space” is reconstructed in the brain by adding eye velocity to retinal slip velocity. But Figure 1 doesn’t execute that analogy at all.

Specific comments:

The mechanical properties of the motor system make the actual stimulus a little messy compared to the desired stimulus, but this issue is mitigated by the careful measurement of actual motor movement.

In Figure 4, I am having a hard time discerning whether the changes in model parameters are “systematic” in trials 15-30. I can see that they changed from 1 to 15, but then they seem much more variable to me. Perhaps this isn’t the best way to present the data given the thinness of the curves and the challenge of discriminating and identifying exact colors. I do agree based on Figure 5 that the model gave a good account of the actual responses.

I need an intuition for why the frequency with the peak amplitude seems to move the left across trials in Figure 4, but the center frequency of the model does not seem to change in Figure 6. I also could benefit from an intuitive reminder of what the “quality factor” means at this stage of the paper, even though it is fairly clearly stated in the modeling section of the Methods.

I am curious whether the “mean absolute error” (Figure 7) is fairly high mainly because of delays in the movement relative to the stimulus, or if it comes from failures to follow certain frequencies. I also am curious whether there is any theoretical basis for thinking about the optimal size of the auditory fovea - would 7 deg be adequate to optimize auditory processing of the cues that yield location?

I would like to see the Abstract come to the point of what is contributed by this study more quickly, with less emphasis on the past.

Reviewer #2

The manuscript is well motivated both in terms of aiming for more naturalistic conditions in laboratory experiments, and in the parallels and motivations it draws from the visual system to put forward its hypothesis in the auditory system.

By asking their subjects to move the head in pursuit of an auditory stimulus, they show that auditory object smooth pursuit is doable by humans and provide indirect support for the notion of an auditory fovea.

Overall, it is a step forward in the understanding of sensory perception in realistic conditions. Especially compared to traditional, head fixed experiments.

The study pushes for more realistic conditions in the study of sensory biology, and in doing so shows interesting results. I also find it to be tight and well-written, which I celebrate. I have only a few concerns, which I describe below:

Major concerns

-How much did the subjects’ head orientation deviate from the horizontal plane? And how much head tilting did they do? If subjects deviated the center gaze from the stimulus plane, or even if not, but they tilted their head at times, then the stimulation would not fall in the horizontal plane, which may affect the auditory processes taking place. Ideally, this control should be shown, and if the stimulation did deviate from the horizontal plane, its likely effect on the interpretation of the results should be made explicit.

-In the discussion, line 463, the authors discuss several possible reasons for the systematic change in the damping coefficient Q. The first one, response accuracy, they show did not change across trials, so it seems unlikely. The second one proposed, movement effort, is a variable the authors do have available in the data (such as: total head displacement, number of head turn events, etc.), and could test whether it changed across trials. Figure 8 shows the model/simulation results of this, which is encouraging, but it would be valuable if the authors could test whether they can extract similar results from the actual behavior and not just from the model.

-Calculated pursuit performance is constant across trials. Was it also calculated within each trial? With a sliding window for example? I think it would be interesting to see if besides the lack of ’learning’ or long-term performance changes, there is a short term intra-trial performance change.

Minor suggestions

-The stimuli frequency range will elicit both ILD and ITD systems. Can a short discussion be added on whether one or the other has a greater relevance for the results?

-For future experiments, it would be useful to know what noise did the rotating motor generate and what was its intensity. In other words, what was the intensity difference between the motor and the stimuli at the position of the microphone?

-The discussion says that the change in Q parameter is an adaptation to the learned stimulus characteristics. Any stimulus characteristic specifically? Even if speculative, this would help and inspire future directions.

-In line 351: ’we obtained similar results with other participants’. This would be nice to see. Perhaps a new panel in figure 4 with the average curves for all subjects?

-In line 358 and/or figure 5: It would be interesting to also report at least the median r2 per subject. To get an idea of how conserved this result is across subjects.

Line 12: ‘impossible’ is too strong. I recommend toning it down.

Line 17: ‘Revealing’ would be an experimental result. Perhaps change for ’suggesting’

Line 70: The grammar is a bit off. Maybe a word missing?

Line 161: What was the aluminum rod’s weight?

Line 163: ‘...was be projected...’ presumably a grammar mistake?

Line 175: ‘in’ not ‘into’.

Line 338: ‘suggest’ to ‘suggests’

## Author Response

March 03, 2021

Dear Prof. Maler,

We were pleased with the constructive and insightful remarks of the two reviewers, which have greatly helped us

to improve our manuscript. On the basis of their criticisms and suggestions, we have updated the text

substantially in the Abstract, Introduction and Discussion, wherever appropriate, and have updated the Figures,

as requested. We also updated and clarified some of the equations used in the modelling, and data analysis.

In addition, we performed new analyses on our data, motivated by both reviewers, which have now also been

included in the revised manuscript. In short, we updated Figure 1 (new conceptual scheme in the Introduction),

Figure 4 (we added the mean results of all subjects), Figure 7 (use of the true head-movement delays), Figure 8

(new figure with an analysis supporting the idea that the head movements are driven by both head-centered

velocity error, and by position error, and summarized in Table 1), Figure 9 (we added panel C with an analysis of

movement effort from the actual data), and Figure 10 (a slightly different feedback model).

We also removed the Appendix, as requested.

Below you will find the detailed responses (in red font) to all issues raised by the reviewers.

We hope that our manuscript is now acceptable for publication in eNeuro,

on behalf of the co-authors,

the corresponding author

Rebuttal:

--------------------------------------------

Manuscript Instructions

- Journal does not publish appendices. Please remove or incorporate into text of manuscript.

Appendix is removed.

- The species studied is not mentioned in the abstract. Please make sure to update both the abstract in the article

file and on the submission form.

The species (human) is now mentioned in the Abstract.

---------------------------------------------

Reviewer #1

This is a sound study that provides new data and an interesting insight into tracking of moving auditory stimuli. It

has multiple strengths and two serious flaws, both fixable.

We thank the reviewer for the constructive criticisms.

Strengths:

(1) the modeling framework seems sound and the data are new, clean, and properly analyzed; (2) the concept of

an “auditory fovea” is new for me and it comes from the revelation (also new for me) that the head (rather than

the eyes) should smoothly track moving auditory stimuli; (3) the adaptation of system performance across trials is

interesting and makes sense.

Weaknesses:

The first flaw is the conclusion that the head tracks auditory target velocity and therefore that there must be an

auditory motion system in the brain as there is for visual motion. But the study presents no evidence that the

head is tracking motion. It could as easily be tracking auditory position and neither critical data nor critical

analysis is presented to discriminate these two possibilities. For eye pursuit, it is the step-ramp of Rashbass

(1961!) that proves the system is tracking motion, but that kind of experiment probably isn’t going to work for

head tracking of auditory stimuli. I think the authors just need to back off here.

The reviewer correctly remarks that we did not prove the existence of an auditory velocity-sensitive system that

operates in a feedback way to minimize auditory slip velocity. We have trimmed down our statements on this in

the Abstract, Introduction, and Discussion.

We based our experiments and rationale in the Introduction, by considering a potential analogy between the

visual and auditory systems (eye movements in response to visual target motion and retinal position changes, vs.

head movements in response to auditory motion and craniocentric position changes), as well as with 2

neurophysiological evidence obtained from several animal species that hint at velocity sensitivity of neurons in

the auditory system at both the midbrain (Inferior Colliculus) and cortical levels.

We think that our data would support such a mechanism. The Rashbass step-ramp experiment (well-known from

the visuomotor literature), or some adapted version of it, is indeed an interesting paradigm, which we will

certainly consider in our follow-up studies.

However, we suggest that the random motion stimulus can in principle dissociate position errors and

velocity-elicited responses. To look into this, we performed an additional analysis on our data by assessing the

relations between the instantaneous head position and the localization position errors vs. a regression on head

velocity vs. the head-centered velocity errors. To that end, we optimally aligned the head-movement responses

with the stimulus motion by minimizing the mean absolute error during the trial (as described for Fig. 7). This

analysis (now highlighted in the new Figure 8) shows that the velocity regression and the position regression both

give good predictions for the behavior (a correlation of 0.63 and 0.69, respectively, for more than 725000 data

points). The head velocity was unrelated to instantaneous target position (r = 0) and, of course, also the position

error and velocity error were mutually unrelated (r = -0.01). We summarized eight linear regression results on the

head-velocity and position data in Table 1. The results suggest that sound position and velocity may be

processed by separate mechanisms: one that responds to position error and one that responds to velocity slip.

Both signals are required to bring (position) and keep (velocity) the head near the ’auditory fovea’. From these

additional results we now cautiously conclude that the responses were at least partially driven by craniocentric

sound-velocity error too.

The second flaw is the structure of Figure 1 and this seems like a fundamental issue. It does not invalidate the

study, but it fails to provide a proper conceptual framework and needs to be fixed. The auditory brainstem should

be sensing sound velocity relative to the (moving) head, which will be auditory slip velocity by the authors’

terminology. I don’t see exactly how to fix this other than to feedback head velocity all the way to the beginning of

the diagram. Also, I think that the authors are trying to make an analogy to the Robinson view of the visual

smooth pursuit system where he proposed that “target velocity in space” is reconstructed in the brain by adding

eye velocity to retinal slip velocity. But Figure 1 doesn’t execute that analogy at all.

The reviewer is correct, and pointed out an obvious mistake on our side. The figure clearly required an update,

as indeed the moving head changes the auditory cues of a moving (or stationary) sound source (in the same way

as the moving eye changes the retinal motion of a moving or stationary visual stimulus), and therefore

automatically creates sound-motion information re. head. This was not as such indicated in the original Figure 1.

Thus, changes in ILD and ITD cues do not signal sound velocity, but sound velocity and changes in sound

position relative to the head (which determines the “auditory slip error” in velocity and position). We have now

changed this figure in line with our original intention. It is now also more in line with the closed-loop nature of a

potential auditory pursuit system (where the head movement directly feeds back to the sensory input), in much

the same way as in Robinson’s (simplified) equivalent for visual pursuit, where the eye movement feeds back to

the visual input.

Specific comments:

The mechanical properties of the motor system make the actual stimulus a little messy compared to the desired

stimulus, but this issue is mitigated by the careful measurement of actual motor movement.

Indeed, in our analysis we used the actual motor output to describe the stimulus motion.

In Figure 4, I am having a hard time discerning whether the changes in model parameters are “systematic” in

trials 15-30. I can see that they changed from 1 to 15, but then they seem much more variable to me. Perhaps

this isn’t the best way to present the data given the thinness of the curves and the challenge of discriminating and

identifying exact colors. I do agree based on Figure 5 that the model gave a good account of the actual

responses.

Figure 4 serves to illustrate the fits for all individual trials of a single subject. A general pattern in the overall color

gradient is visible. However, the data (and the associated model parameters) are endowed with considerable

trial-to-trial variability, which makes it hard to assess a trial-to-trial trend from beginning to end solely on the basis

of this color scheme. Note also that the model contains more than one parameter, and from the shape of the

curves alone it is not immediately obvious which of these parameters changes in a systematic way with trial

number, as both parameters have an effect on the shape of the amplitude characteristic. That’s why in Figure 6

the regression analysis is much more robust and reliable for such an assessment. Although there we show the

data from a different subject (side note: in a previous submission we were criticized for showing exemplary data

from the same subject...), these results were quite typical, as can also be judged from the pooled results in Fig.

6C,D.

I need an intuition for why the frequency with the peak amplitude seems to move to the left across trials in Figure

4, but the center frequency of the model does not seem to change in Figure 6. I also could benefit from an

intuitive reminder of what the “quality factor” means at this stage of the paper, even though it is fairly clearly

stated in the modeling section of the Methods.3

Indeed, as explained above, this is not immediately intuitive, as the shape of the gain characteristic depends on

Q as well as on the (undamped) resonance frequency, wC. Eqn. 9 summarizes these features. From this

equation it can be appreciated that when the center (resonance) frequency does not change, but the quality

factor decreases, the location of the peak will shift to lower frequencies. At the same time, the amplitude, which

solely depends on Q, will decrease too (we have now also added this equation). Note that these properties are

also illustrated in the step responses of Fig. 9.

I am curious whether the “mean absolute error” (Figure 7) is fairly high mainly because of delays in the

movement relative to the stimulus, or if it comes from failures to follow certain frequencies. I also am curious

whether there is any theoretical basis for thinking about the optimal size of the auditory fovea - would 7 deg be

adequate to optimize auditory processing of the cues that yield location?

We made an mistake in these calculations by taking the fixed internal delay, TD, instead of the true head movement delay (which also adds the considerable phase shift introduced by the 2nd

-order characteristic). We

have therefore modified this analysis slightly by replacing the model’s fixed added delay, TD, which varied

between 15-95 ms among participants, by an optimal trial-by-trial estimate for the total mean head-movement

delay, which varied between about 220 and 650 ms. To that end, we performed a brute search for each trial, in

which we calculated for every possible delay between 0 and 1.0 s, the mean absolute error of the trial by Eqn.

10. We then selected the delay that yielded the smallest MAE, and called it the optimal delay, Topt for that trial.

The new Fig. 7 shows the absolute errors obtained at these delays, which now scatter around 5 deg. The trend is

the same: the error does not change with trial number. The errors are relatively high because the auditory system

could not (learn to) make any predictions about the stimulus movement. A second contribution to the error is

failure of the system to follow the higher stimulus-movement frequencies with a gain of 1.0 and a phase of 0.0

deg.

I would like to see the Abstract come to the point of what is contributed by this study more quickly, with less

emphasis on the past.

We removed the initial phrases of the abstract, and have now put more emphasis on our new findings.

---------------------------------------------

Reviewer #2

The manuscript is well motivated both in terms of aiming for more naturalistic conditions in laboratory

experiments, and in the parallels and motivations it draws from the visual system to put forward its hypothesis in

the auditory system.

By asking their subjects to move the head in pursuit of an auditory stimulus, they show that auditory object

smooth pursuit is doable by humans and provide indirect support for the notion of an auditory fovea.

Overall, it is a step forward in the understanding of sensory perception in realistic conditions. Especially

compared to traditional, head fixed experiments.

The study pushes for more realistic conditions in the study of sensory biology, and in doing so shows interesting

results. I also find it to be tight and well-written, which I celebrate. I have only a few concerns, which I describe

below:

We thank the reviewer for these encouraging comments.

Major concerns

-How much did the subjects’ head orientation deviate from the horizontal plane? And how much head tilting did

they do? If subjects deviated the center gaze from the stimulus plane, or even if not, but they tilted their head at

times, then the stimulation would not fall in the horizontal plane, which may affect the auditory processes taking

place. Ideally, this control should be shown, and if the stimulation did deviate from the horizontal plane, its likely

effect on the interpretation of the results should be made explicit.

Deviations of the head movements from the horizontal plane were very small. Sounds were broadband and well localizable (which we tested in a separate 2D localization control experiment). Moreover, subjects knew (from

having seen the setup) that stimulus motion of the robot was confined to the horizontal plane, and although not

specifically instructed as such, their responses showed hardly any vertical displacements. We now made a note

of this early on in the Methods.

-In the discussion, line 463, the authors discuss several possible reasons for the systematic change in the

damping coefficient Q. The first one, response accuracy, they show did not change across trials, so it seems

unlikely. The second one proposed, movement effort, is a variable the authors do have available in the data

(such as: total head displacement, number of head turn events, etc.), and could test whether it changed across 4

trials. Figure 8 shows the model/simulation results of this, which is encouraging, but it would be valuable if the

authors could test whether they can extract similar results from the actual behavior and not just from the model.

The reviewer made a very interesting proposal, which we gladly took up for further investigation. As shown in

Figure 5A,B, the model parameters provided a very good description for the movement data of individual trials.

We therefore decided to characterize “total movement effort” on the basis of these model fits, as the stimulus

movements themselves were pseudo-random and thus varied considerably from trial to trial. Thus, an analysis in

the time domain, like determining the number of head-turns per trial, or similar, would not be a good, unbiased

measure. We therefore determined, for each trial, the fitted gain characteristics from the actual fit-parameters of

the model, and calculated the effort from these characteristics by the mean total spectral power (now added to

Eqn. 12) as function of trial number. In the new Figure 9 (panel C), we plotted the estimated power for all trials

and all 11 subjects. The results show that this measure of effort indeed decreased during the course of the

experiment by 34%, with an overall correlation of r=-0.78. We have now incorporated this analysis in the

Discussion, and thank the reviewer for this valuable suggestion.

-Calculated pursuit performance is constant across trials. Was it also calculated within each trial? With a sliding

window for example? I think it would be interesting to see if besides the lack of ’learning’ or long-term

performance changes, there is a short term intra-trial performance change.

As the stimuli were relatively low-frequency (0.05 to maximum 1.05 Hz, trial duration 20 s), windowing as

suggested by the reviewer may prove tricky. However, to give an idea, we performed such an analysis, in which

we averaged the mean absolute error in 145 overlapping windows of 200 ms, with 50 ms overlap. We did not

observe any trend of the error with time. The behavior of the error was random and did not differ between the first

and the last trial.

Mean absolute errors as function of time during each trial (averaged over a 200 ms wide sliding window), shown

for the first (black dots) and the last (red dots) trial of the experiment for each subject (S1-S11). Blue thin lines:

linear regressions for all 30 trials of each subject. Although the error varies, the changes are not systematic, and

do not differ between trials. The slopes of the lines scatter around zero.

Minor suggestions

-The stimuli frequency range will elicit both ILD and ITD systems. Can a short discussion be added on whether

one or the other has a greater relevance for the results?

We did not test this explicitly in our experiments, so we do not know (yet) whether low-pass filtered noises (ITD

pathway) vs. high-pass filtered noises (ILD pathway) vs. broadband (as used here) would yield different results.

This interesting point is something for future work, and we have now added a remark on this in the Discussion.

-For future experiments, it would be useful to know what noise did the rotating motor generate and what was its

intensity. In other words, what was the intensity difference between the motor and the stimuli at the position of

the microphone?

The buzzing sounds coming from the activated motor were about 50 dBA at the subject’s ears, and always came

from the subject’s zenith, 90 deg away from the horizontal plane. These sounds did not provide any stimulus

location or directional cue. We tested this qualitatively with the motor sounds on without a target sound. When

the target sound played, the motor sounds did not interfere with the listeners’ sound-localization abilities. This

statement has now also been included in the Methods.

-The discussion says that the change in Q parameter is an adaptation to the learned stimulus characteristics. Any

stimulus characteristic specifically? Even if speculative, this would help and inspire future directions.

We think that the system acquired knowledge about the frequency spectrum of the movements. To verify that this

is indeed the case, future experiments could manipulate the amount of consistent spectral information in the

trajectories. For example, if the spectral content would vary randomly from trial to trial, the amount of adaptation

in the Q-factor might be reduced. This remains to be tested. We included a remark on this in the Discussion.

-In line 351: ‘we obtained similar results with other participants’. This would be nice to see. Perhaps a new panel

in figure 4 with the average curves for all subjects?

We have now added inset panels for the gain and phase characteristics with the mean results from all 11

participants.

-In line 358 and/or figure 5: It would be interesting to also report at least the median r2 per subject. To get an idea

of how conserved this result is across subjects.

The mean std of the r2

values across 11 subjects was 0.83 {plus minus} 0.10, and is now included in the text.

Line 12: ‘impossible’ is too strong. I recommend toning it down. replaced by: ’poor’

Line 17: ‘Revealing’ would be an experimental result. Perhaps change for ’suggesting’ done

Line 70: The grammar is a bit off. Maybe a word missing? changed

Line 161: What was the aluminum rod’s weight? about 50 g, added in the text.

Line 163: ‘...was be projected...’ presumably a grammar mistake? changed

Line 175: ‘in’ not ‘into’. changed

Line 338: ‘suggest’ to ‘suggests’ changed
